# Communication calls produced by electrical stimulation of four structures in the guinea pig brain

**DOI:** 10.1371/journal.pone.0194091

**Published:** 2018-03-27

**Authors:** David B. Green, Trevor M. Shackleton, Jasmine M. S. Grimsley, Oliver Zobay, Alan R. Palmer, Mark N. Wallace

**Affiliations:** 1 Medical Research Council Institute of Hearing Research, School of Medicine, The University of Nottingham, Nottingham, United Kingdom; 2 Department of Anatomy and Neurobiology, Northeast Ohio Medical University, Rootstown, Ohio, United States of America; Universidad de Salamanca, SPAIN

## Abstract

One of the main central processes affecting the cortical representation of conspecific vocalizations is the collateral output from the extended motor system for call generation. Before starting to study this interaction we sought to compare the characteristics of calls produced by stimulating four different parts of the brain in guinea pigs (*Cavia porcellus*). By using anaesthetised animals we were able to reposition electrodes without distressing the animals. Trains of 100 electrical pulses were used to stimulate the midbrain periaqueductal grey (PAG), hypothalamus, amygdala, and anterior cingulate cortex (ACC). Each structure produced a similar range of calls, but in significantly different proportions. Two of the spontaneous calls (chirrup and purr) were never produced by electrical stimulation and although we identified versions of chutter, durr and tooth chatter, they differed significantly from our natural call templates. However, we were routinely able to elicit seven other identifiable calls. All seven calls were produced both during the 1.6 s period of stimulation and subsequently in a period which could last for more than a minute. A single stimulation site could produce four or five different calls, but the amygdala was much less likely to produce a scream, whistle or rising whistle than any of the other structures. These three high-frequency calls were more likely to be produced by females than males. There were also differences in the timing of the call production with the amygdala primarily producing calls during the electrical stimulation and the hypothalamus mainly producing calls after the electrical stimulation. For all four structures a significantly higher stimulation current was required in males than females. We conclude that all four structures can be stimulated to produce fictive vocalizations that should be useful in studying the relationship between the vocal motor system and cortical sensory representation.

## Introduction

Animal vocalizations are thought to be under the control of two distinct but overlapping motor systems: the first produces innate calls that are stereotypical and relatively invariant across any one species and does not involve any input from the motor cortex; the second produces more variable calls or songs which may involve a unique recombination of basic syllables, or modulation of calls, and that is under the conscious control of cortical motor pathways [1]. The degree to which a particular species can learn to change its pattern of vocalizations remains controversial, but, although there has been some evidence of reinforcement learning by intracranial stimulation of the hypothalamus in the guinea pig [2], there has not yet been any evidence for a role of the motor cortex. Even in primates (excluding the great apes), vocalizations are thought to be largely innate and most are related to the affective/motivational state of the caller [3]. The current study investigates the innate, automatically produced calls.

The guinea pig a very social animal and is a suitable small mammal for the study of the production and perception of conspecific vocalizations. Between 11 and 13 calls have been identified in the guinea pig and the behavioural context of the calls has been identified both in the wild and in large caged colonies [4–6]. Some of these calls seem to be consistently produced in a reflex response to a particular situation. Thus, a prolonged series of isolation calls is produced when infants (less than about three weeks old) are separated from their mother [7], while the rhythmic durr can be reliably produced by a mildly startling sound in both juveniles and adults [8] and tooth chatter is produced in response to an external threat [9]. More recently, we collected and analysed these basic calls with modern digital analysis methods and used them as stimuli for analysing the responses of neurons in the auditory cortex [10]. Here, as a prelude to studying the interaction between the vocal production pathways and the sensory representation [11], we sought to determine whether different brain structures were associated with specific calls, so that we could produce a call by electrical stimulation of a particular structure. Electrical brain stimulation has been used in both awake [12, 13] and anaesthetised [12, 14–16] guinea pigs to produce calls. These studies have led to the suggestion that particular calls may be associated with certain brain areas more than others [12, 14, 17]. All the innate calls should be produced by the premotor cells located in and around the periaqueductal grey matter (PAG) of the midbrain and pons [18] as this structure is thought to be a final common pathway for forebrain structures involved in producing innate calls. The initiation of isolation calls is apparently under the control of the anterior cingulate cortex (ACC) which has a strong descending input into the lateral PAG [1]. The hypothalamus (hypo) is another important area in generating automatic calls and it has been suggested that it may be particularly important for initiating calls, such as the purr [17], which are associated with mating behaviour [5], but are not produced by stimulation of the PAG [16]. There is also evidence that the amygdala is involved in producing some calls, either directly [12, 14, 19], or as part of a limbic network that also involves the hypothalamus [20]. Thus, all four of these brain structures were separately stimulated in an attempt to understand more about the role of each in call production.

## Materials and methods

### Surgical preparation

A total of 22 pigmented guinea pigs of both sexes and weighing 257–1255 g were used in this study. All animals had been weaned and the youngest two were 23 and 27 days old at the time of data collection. All the others were between 1 and 24 months old (see Table 1). Anaesthesia was induced by an intraperitoneal injection of 20% urethane (Sigma) solution at 2.5 ml/kg and supplemented, as necessary, by intramuscular injections of ketamine/xylazine (3:2 by volume), (Ketaset/Rompun) to abolish the forepaw withdrawal reflex during the surgery. Ketaset (Fort Dodge Animal Health) contained Ketamine at 100 mg/ml while Rompun (Bayer) contained xylazine at 20 mg/ml. Atropine sulphate was given subcutaneously shortly after the onset of anaesthesia, 0.2 mg/kg, (Hameln pharmaceuticals).

**Table 1:** Summary of structures stimulated and calls produced.

Expt #	Sex	Age (mths)	Weight (g)	Structure stimulated	#stimulus trains	# of calls	Calls/ train	Mean current (μA)
1123	F	5	816	ACC	22	390	18	432
1127	F	2.5	653	ACC	15	275	18	237
1130	M	2	717	ACC	12	253	21	454
1133	M	2	752	ACC	29	496	17	375
1151	M	1	342	ACC	3	22	7	600
1135	F	2.5	585	Hypo	73	1823	25	63
1144	M	1.5	521	Hypo	74	1366	18	92
1160	F	1	374	Hypo	45	804	18	88
1162	M	23	1207	Hypo	4	81	20	50
1167	F	19	1255	PAG	61	966	16	132
1168	F	24	1061	PAG	47	1316	28	164
1170	M	3	925	PAG	3	27	9	123
1198	M	3.5	857	PAG	6	23	4	333
1202	F	9	1063	PAG	12	274	23	179
1208	F	14	1100	PAG	111	1362	12	98
1210	F	1	257	PAG	99	3697	37	102
1227	F	1.25	593	PAG	173	1245	7	80
1173	M	3.5	928	AMYG	32	1331	42	252
1179	F	4.5	891	AMYG	39	175	4	126
1193	M	3	870	AMYG	189	1015	5	276
1194	M	3.5	898	AMYG	249	874	4	238
1230	M	0.75	285	AMYG	74	506	7	249

Core temperature was maintained at 38°C by a heating blanket (Harvard) and rectal probe. A midline incision was made, four burr holes made in the rostral skull and small stainless screws inserted. A metal bar was then attached to the skull with dental acrylic so that the head could be fixed during surgery. A craniotomy was performed above the appropriate brain regions, and the dura removed. Electrodes were inserted into the brain using stereotaxic coordinates [21]. Electrolytic lesions were made in some of the tracks by passing a current of 100 μA for 10 seconds (electrode negative) through the electrode. At the end of the experiments, the animal was given an overdose of pentobarbitone and perfused with 4% paraformaldehyde before having its brain removed and sectioned on a sledge microtome. All experiments were performed in accordance with the 1986 European Communities Council Directive (86/609/EEC) with the approval of the ethical review committee at the University of Nottingham, UK and under the authority of Home Office Project Licence PPL 40/3616. The design and performance of the experiments was in accordance with ARRIVE Guidelines and their checklist was completed (S1 Table).

### Electrical stimulation

An array of 4 glass-coated tungsten electrodes with thick long tips (100 μm long) (Bullock, 1988) were attached to a printed circuit board. Electrodes were capable of carrying up to 800 μA and were arranged with approximately 100–200 μm between the tips. An isolated pulse stimulator (AM systems) was used to deliver a biphasic square wave pulse train at 60 Hz for 1.6 s to give a train of 100 pulses. Each pulse lasted 1 ms. Stimulation current ranged from 15–800 μA and was bipolar. For each position of the electrode assembly in the brain, each of the four electrodes could be separately assigned as the cathode, and the anode was assigned as the adjacent electrode to form a stimulation pair. Current was never passed down more than one electrode at a time.

### Audio recording of vocalizations

A condenser microphone (Model B-5 Behringer) was used to record spontaneously vocalizing guinea pigs, either in their home cage or in groups of three in a sound attenuating room. Audition 1 software (Adobe) recorded 24 bit stereo, sampled at 48.8 kHz. Electrically elicited vocalizations were recorded using a battery powered Handy Recorder H2 (Zoom) (16 bit, sample rate 96 kHz) placed ~5cm in front of the guinea pig’s mouth.

### Histological verification of call production loci

**Fig 1:**
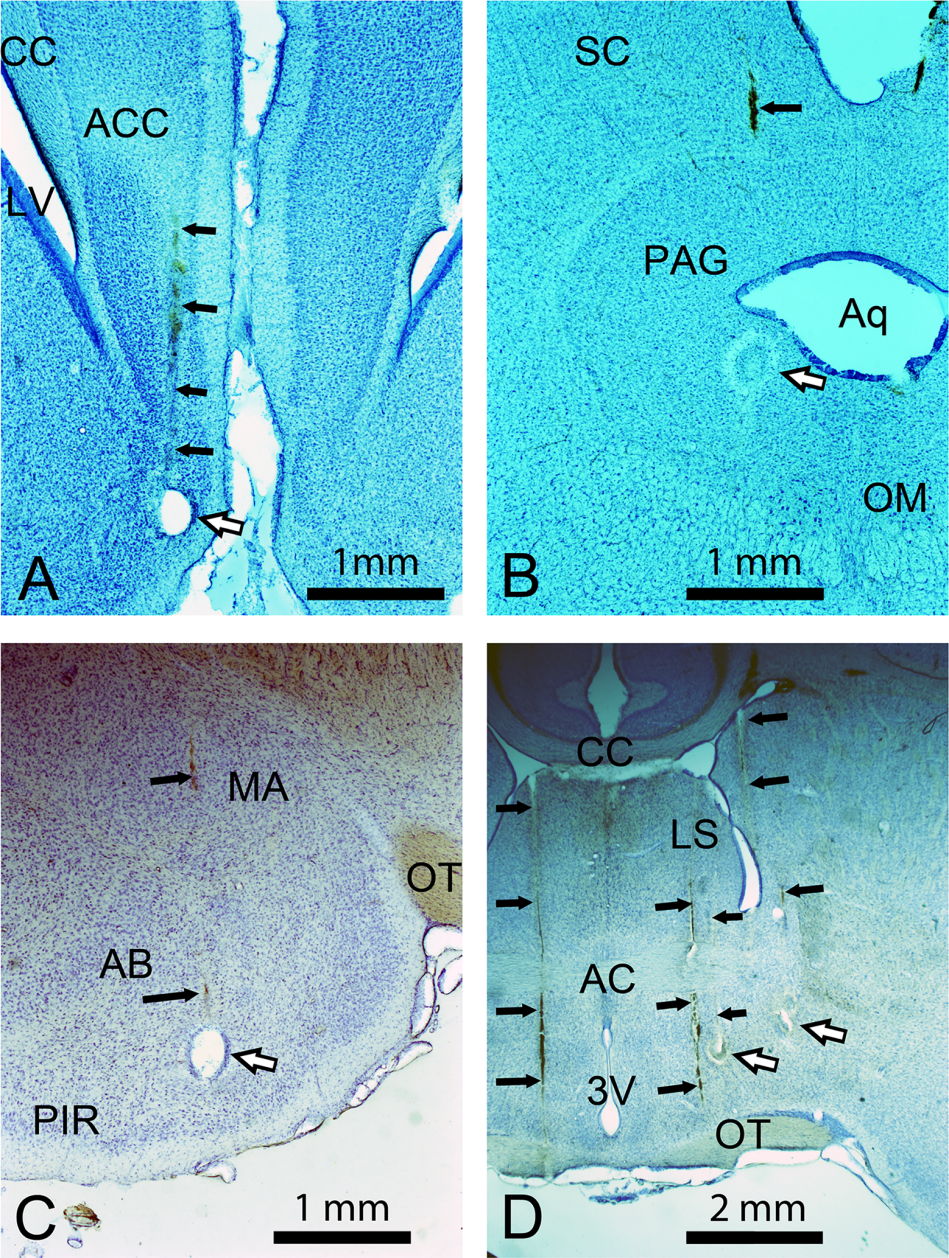
Confirmation of electrode position. Histological examples of electrode tracks and lesions in Nissl stained sections cut in the coronal plane through the four structures examined in this study. Electrode tracks, which usually contained a line of red blood corpuscles, are indicated by the small black arrows while the lesions are indicated by the larger white arrows with a black border. **A** Section through the anterior cingulate cortex with a lesion just ventral to the cortical area which produced communication calls when electrically stimulated. **B** Section through the midbrain showing the edge of a lesion in the peri-aqueductal grey matter. **C** Section through the amygdaloid complex with a lesion at the ventral edge. **D** Section through the forebrain at the level of the anterior commissure with evidence of five electrode tracks two of which end in lesions in the hypothalamus. This section was photographed at a lower magnification than the other three so that all five tracks could be seen. Abbreviations: 3V, third ventricle; AB, basal amygdaloid nucleus; AC, anterior commissure; ACC, anterior cingulate cortex; Aq, cerebral aqueduct; CC, corpus callosum; LS, lateral septum; LV, lateral ventricle; MA, medial amygdaloid nucleus; OM, oculomotor nucleus; OT, optic tract; PAG, periaqueductal grey matter; PIR, piriform cortex; SC, superior colliculus.

The animals used in the microstimulation experiments covered a broad size/weight range which meant that standard stereotaxic coordinates would not always refer to the same position. Thus histological verification of electrode position was made by relating lesion position to structures identified by reference to the guinea pig atlas [21] using landmarks dispersed across the whole section. Fig 1 shows example Nissl stained coronal sections with electrolytic lesions.

### Characterization of vocalizations

Audio recordings of spontaneously vocalising guinea pigs and the electrically elicited calls, were analysed using the same procedure. From a sample of 2335 recordings we first excluded any recordings that were of poor signal to noise ratio or contained interfering noises of any kind. This left 1459 recordings of evoked vocalizations that varied between 2 and 30 s long in response to the electrical stimulation of one of the four brain areas. Further analysis of these waveforms was conducted using SAS Lab Pro bioacoustics software (Avisoft). The first stage of this analysis was to detect when vocalizations were present and this is done using a simple thresholding procedure. The signal-to-noise ratio of the 1459 recordings was not uniform and the threshold level was set by manual adjustment looking at the waveforms on screen. Thresholds were reset for every new experiment and for every new electrode position within an experiment (to allow for variations in experimental set up including, for example, the exact position of the recording microphone). Once this was done, all waveforms from that experiment or electrode position were analysed automatically with no further manual intervention. Avisoft is a syllable based analysis suite detecting and logging the onset time and duration of each individual sound pulse that had an amplitude that exceeded the threshold and had a minimum gap of 100 ms from a preceding pulse.

For identification of the calls, for each detected vocalization pulse, a spectrogram was first computed using a 512 point fast Fourier transform with a moving window of overlapping by 50%. The computed spectrogram was then compared with a set of template spectrograms using a two dimensional correlation. Some leeway in this process was provided by Avisoft computing the correlation for a range of frequency offsets in the template and taking the largest correlation value. Our initial analyses used template spectrograms computed from exemplars of naturally occurring vocalizations from our earlier work [10] identified according to Berryman’s definitions [6]. However, since this was a limited sample and many of the calls were quite variable, we created further templates based on electrically stimulated calls, which were identified based on their similarity to the spontaneous calls identified by Berryman[6]. The records could also contain artefactual non-vocalization sounds: to identify and exclude these we also constructed a series of spectrograms of exemplars of the artefactual sounds. For the final analysis we compared each identified vocalization syllable with 9 “chirrup” template spectrograms, 15 “chut” templates, 6 “chutter” templates, 4 “durr” templates, 3 “low-whistle” templates, 1 “purr” template, 14 “rising whistle” templates, 6 “scream” templates, 7 “squeal” templates, 10 “toothchatter” templates, 4 “whine” templates and 7 “whistle” templates. Any vocalization syllable that correlated with any template higher than 0.65 was given the name of the template with which it had the highest correlation.

### Statistical analysis

The data for each brain area came from at least four animals, usually with two female and two male (see Table 1) and a range of weights that correlates with maturity of the larynx and length of the vocal tract. The fact that each animal produced many calls implied a hierarchical structure in the data, which required us to use mixed-effect modelling. With the help of this approach, we tried to disentangle the contributions of site and subject effects to the observed outcomes (call frequencies). Due to its flexibility, it also allowed us to include the effects of varying electrode positions within site for some animals as well as the influence of covariates such as weight or gender. On the other hand, one of the limitations of mixed-effect modelling is that it generally only allows a binary outcome. In practice, this meant that we chose to combine the seven separate identified calls into two classes based on their spectrographic analysis: low-frequency and high-frequency. Some of the calls with primarily low-frequency energy can be expressed in the same behavioural context as the high-frequency calls, but those with high-frequencies are louder and generally express a more intense level of emotion [6].

The following analyses start from a mixed-effect logistic regression model for the probability p_LF_ of low-frequency responses which is defined by

$$log\left(\frac{1-p_{LF}}{p_{LF}}\right)=\beta_{0}+\beta_{Site}+\beta_{Gender}+\beta_{W}*Weight+b_{Subject}+b_{Subject,Position}$$

The term on the left-hand side of this equation is the logit of the high-frequency probability p_HF_ = 1- p_LF_. This is modelled as the sum of an overall average β_0_, the Site effect β_Site_, a Gender effect β_Gender_ and a linear dependence on Weight with slope β_Weight_. The β terms thus describe population-level, subject-independent effects. Variations between animals and between electrode positions within animals are captured by the random-effect terms b_Subject_ and b_Subject,Position_. The fitting procedure provides explicit estimates for the β terms. The two types of random effects are not estimated individually; rather, one only estimates their probability distributions across all animals. (It is assumed that they are “drawn” from normal distributions with mean zero. The fitting procedure estimates the variances of these distributions.) All regressions were performed with the R package lme4: URL https://www.R-project.org/ [22].

## Results

### Identification of calls

**Fig 2:**
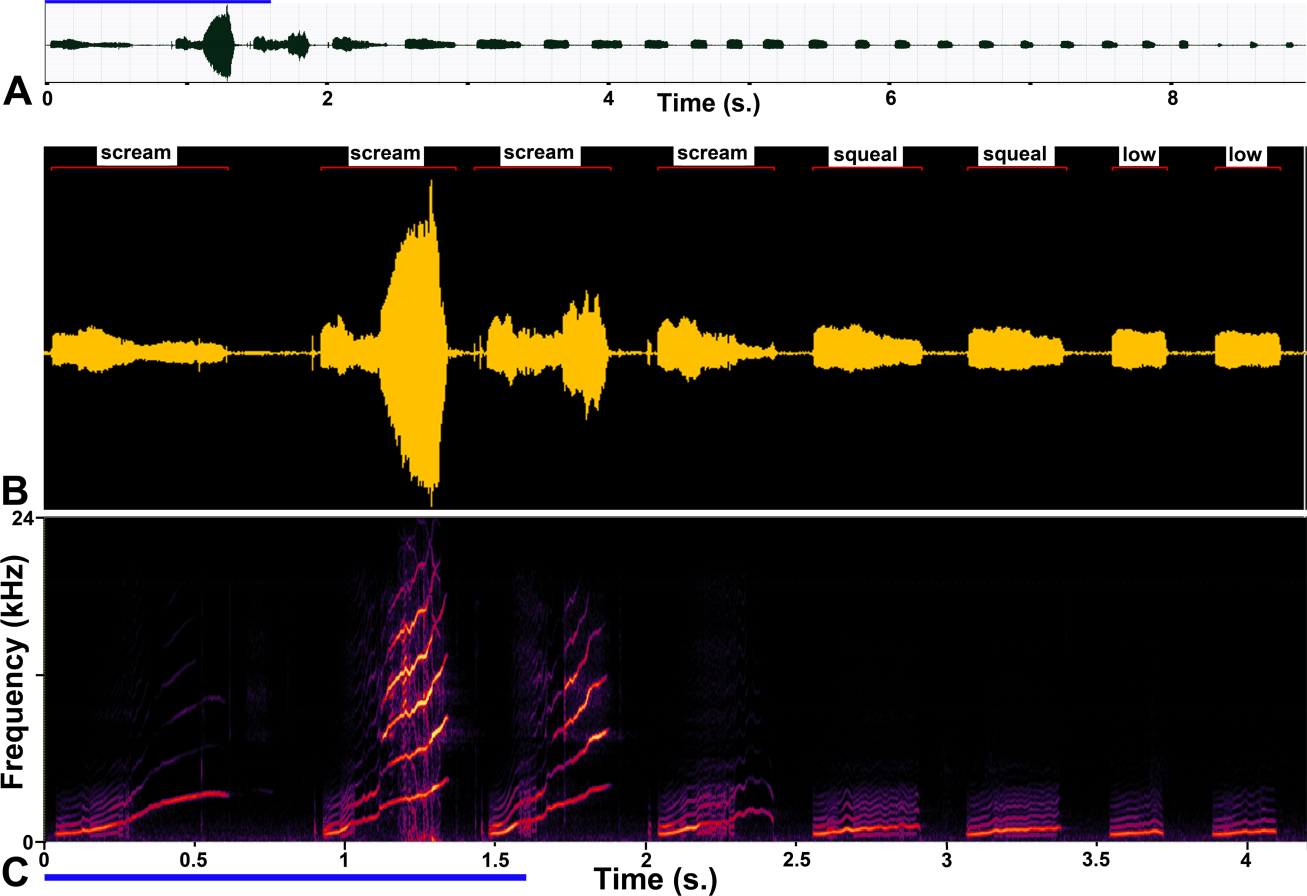
Example of a call sequence produced by stimulating the ACC. **A** Waveform of the sequence which lasted almost 9 seconds. The blue line at the top of the panel represents the electrical stimulus train which lasted for 1.6 seconds. **B** Waveform of the beginning of the call which has been automatically parsed into separate sound pulses by the Avisoft programme. Each sound pulse is identified by a thin red line above which is the name of the call template with which it was most closely correlated. **C** Spectrogram of the sound sequence showing the variation of spectral patterns that could be associated with one call and the gradual change from one call type to another. The blue line at the bottom of the panel represents the stimulus train for this expanded space.

A large number of calls were produced during this study partly because a single stimulus train could produce over a hundred brief calls in a single sequence that could last up to 69 s. The first question to be addressed was to determine objectively how many of them corresponded to the exemplars of spontaneous calls that we had used in a previous study [10]. An example of the classification process is shown in Fig 2. In this figure the sequence of calls produced by stimulation of the ACC is shown in panel A over a period of almost 9 s. The waveform of the calls produced in the first 4 s is shown in Fig 2B with identified vocalization events (after thresholding in the Avisoft programme). Each of these events has been classified by comparing their spectrogram (Fig 2C) with the series of 86 natural templates and a name has been given to the vocalization event. This sample of waveform is very typical of the electrically evoked sounds as it shows a long sequence of evoked vocalizations that start out as one and evolve into others. This can present a difficulty for any classification process as this evolution results in some calls that are intermediate between the classes. For the waveform shown, the first three syllables are clearly quite high frequency, harmonic and moving to higher frequencies (screams) whereas the fifth and sixth are harmonic, have gently rising frequencies and a duration of about 300 ms (squeal) and the last two are only about 200 ms long (low whistle). The 4^th^ in the sequence has been identified as a scream, but has very weak high-frequency components and not much increase in frequency, so is part way to becoming a squeal.

#### Description of natural call exemplars

**Fig 3:**
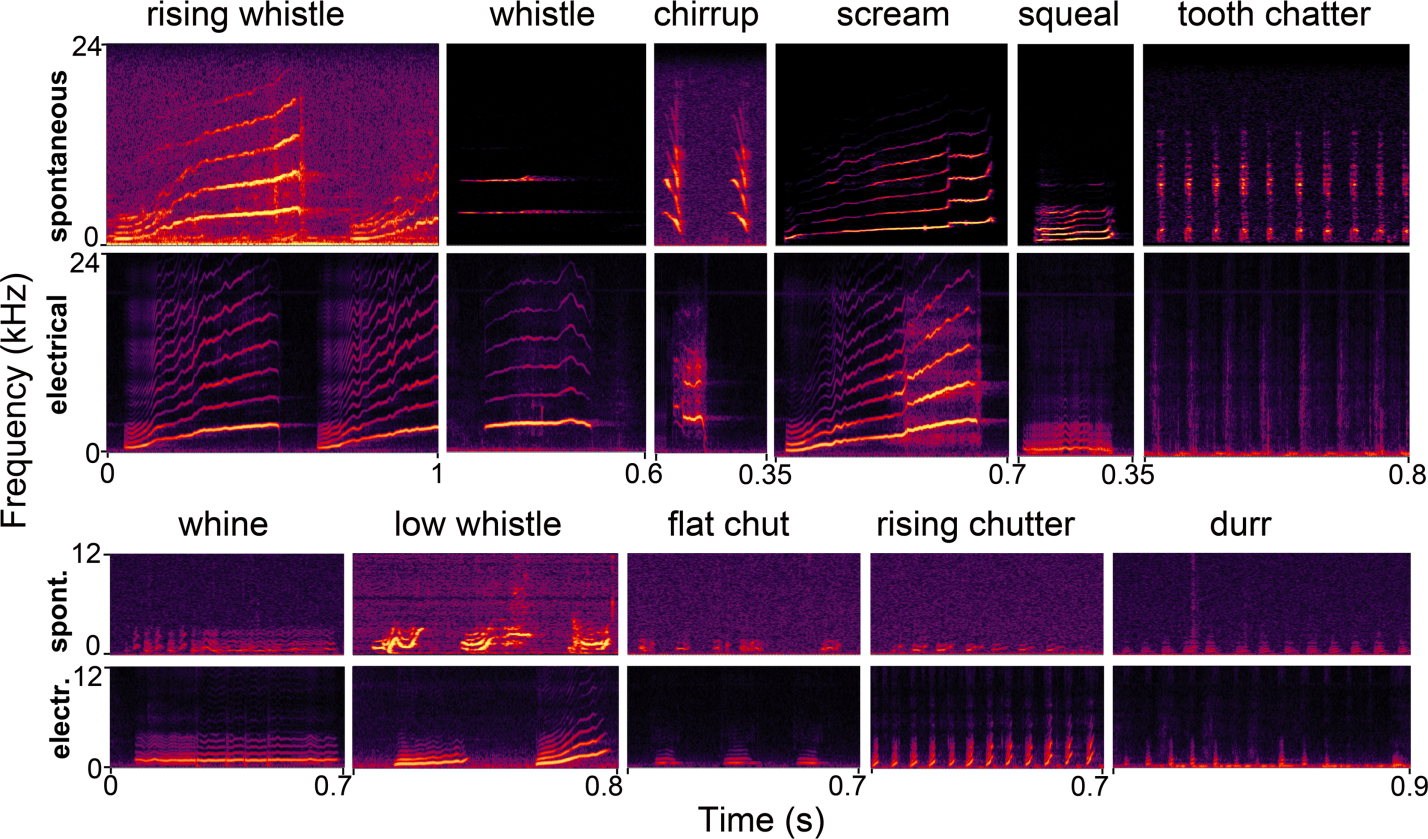
Comparison of natural and electrically produced calls. Example spectrograms of the 11 naturally occurring adult calls that were used as some of the templates for identifying the electrically produced calls in the current study. Each spontaneous call is paired with a spectrogram of an electrically produced call that corresponds to it.

We used exemplars of 10 different voiced calls and 1 non-voiced call (the tooth chatter) based on the definitions of Berryman [6]. These are described below and shown in the upper rows of panels in Fig 3.

1. Rising whistle; harmonic call; fundamental starts at about 500 Hz and after an initial constant frequency phase rises to 3–3.5 kHz before flattening out for a time depending on the duration of the call which varies from 250–1000 ms.
2. Whistle; flat harmonic call with a fundamental of 3 kHz and a duration of 50 to 500 ms.
3. Chirrup; harmonic call with rapidly falling frequency; fundamental starts at 3.5 kHz and falls to 1 kHz over 70 ms; always forming a long regular sequence of calls.
4. Scream; harmonic call with fundamental starting at 800 Hz and rising slowly to about 3 kHz over 500 to 700 ms; often a sudden jump to high frequencies near the end.
5. Squeal; harmonic call with gently rising frequency; fundamental starts at about 500 Hz and rises to 600 to 1000 Hz over a period of 250 to 750 ms.
6. Tooth chatter; brief broadband noise bursts of 25 ms each at 12–14 Hz.
7. Whine; harmonic call with fundamental starting at 400–600 Hz, fairly flat but with small fluctuations in frequency; variable duration of 150–600 ms long.
8. Low whistle: harmonic call with fundamental starting at 500–700 Hz and ending below 3 kHz; modulated up and down in frequency over about 200 ms
9. Chutter; harmonic call with fundamental starting at 400–500 Hz; can be either flat or rising in frequency; each sound pulse lasts about 50–100 ms and they come in rhythmic sequences of 8 or more.
10. Chut; harmonic call with the same characteristics as chutter except they form an irregular sequence or may occur singly.
11. Purr; fundamental frequency of 0.27–0.4 kHz, series of regular pulses of about 50 ms at 13–18 Hz; the durr (short purr) lasts for up to 800 ms; the purr lasts over 1 second.

### Similarity between natural and electrically stimulated calls

A total of 20,977 sound pulses were analysed with the Avisoft software and 18,321 were classified as one of the innate calls based on their correlation value being over 0.65 for at least one of the 86 call templates used in this study. All of the identified calls were similar to one of three high-frequency natural calls: rising whistle, whistle and scream or to the five low-frequency calls: squeal, whine, low whistle, chut and durr. There were no clear matches with the exemplars of chirrup, chutter, purr or tooth chatter. The chirrup is a very distinctive call with a long sequence of downward modulated, high frequency chirps. We never encountered this distinctive pattern following electrical stimulation although there were a few examples where there were quiet sounds with a similar range and duration to an individual pulse of the chirrup call (Fig 3). Chut was the most common call produced by stimulation. There were regular series of chuts which were similar to the chutter, but the inter-pulse interval or spectral range of the individual pulses did not match any of our chutter templates. In the electrical chut sequences there were clear differences between the pulses with some having a fundamental frequency trajectory that was flat, and others where the fundamental was modulated upwards, to give either flat or rising chuts. A few calls (2; 0.01%) did correspond to our durr template, but even these two calls didn’t sound like a natural call and had a high-pitched and artificial quality. The same was true of the tooth chatter as only a few examples (3; 0.014%) had a good correlation with our templates. The stimulated tooth chatter had a slower rate and a less sharp onset than the natural call. Some 1995 (9.5%) sound pulses were classified as artefacts (e.g. miscellaneous laboratory sounds) and 661 (3.15%) were unclassified. Some of the artefacts included tooth chatter, but these chatters had longer pulses of sound and were at a slower or more irregular rate than in the awake examples of tooth chatter. Thus, although there were examples of tooth chatter, they were not sufficiently similar to the naturally occurring templates to be identified as such by the programme. We made no attempt to identify the number of instances of tooth chatter as we weren’t confident of being able to distinguish a tooth click from other types of click. In summary then, the electrical stimulation produced single calls such as low whistle, chut or whine that were very similar to the natural calls, but it did not produce clear examples of the rhythmic calls that occur in long sequences such as chirrup and purr (not present), or chutter and tooth chatter which looked and sounded artificial.

### Presence of juvenile calls

**Fig 4:**
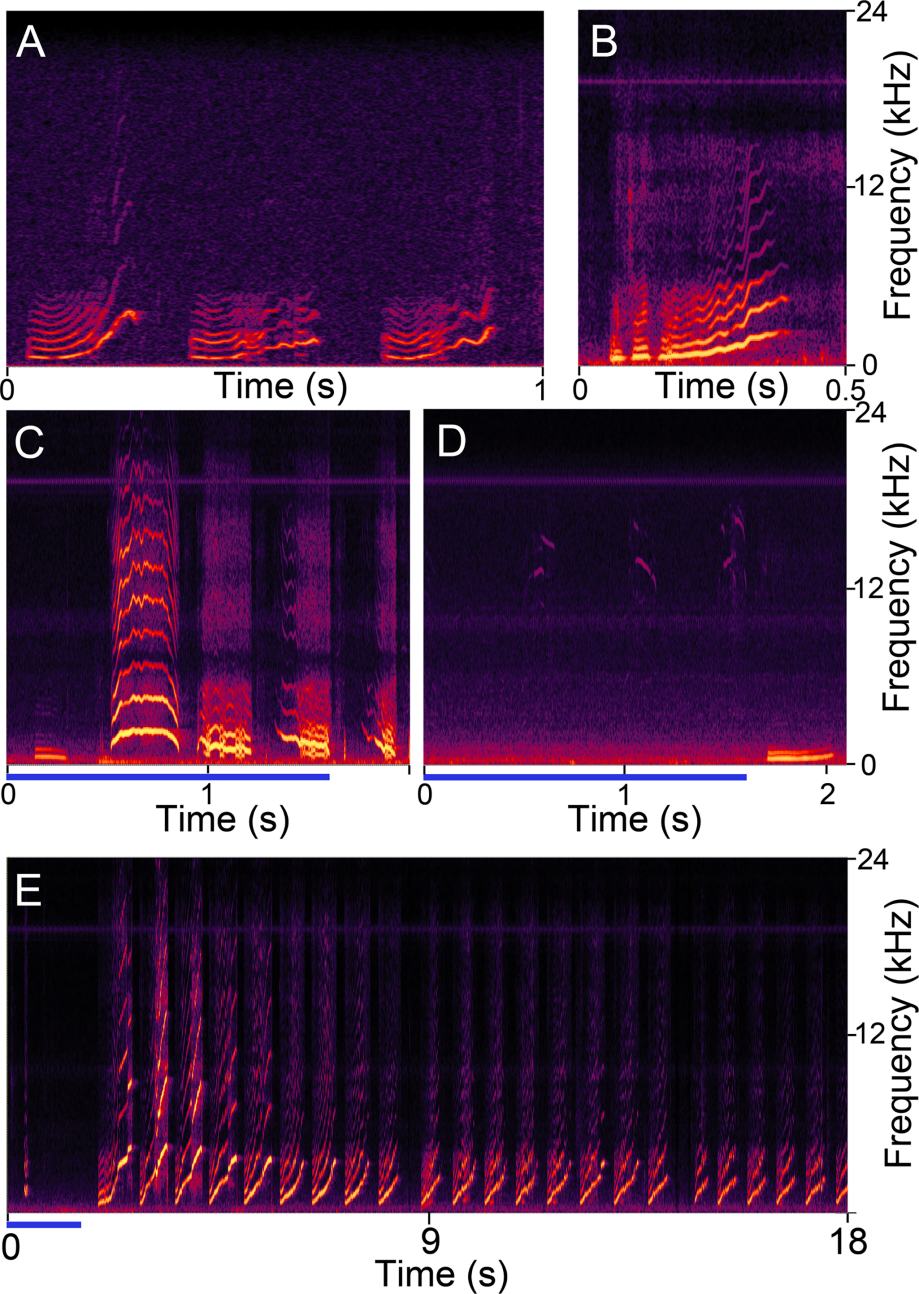
Spectrograms of calls produced by an isolated infant and following electrical stimulation in juvenile animals. **A** Three isolation whistles produced by a young animal (16 days old) separated from its mother and placed by itself in a recording booth. **B** Similar call to those in A produced about 0.4 s after stimulating the amygdala of a 23 day old animal. **C** Further examples of distinctive juvenile calls produced by amygdalar stimulation in the same animal as shown in B. During the stimulus train (blue line at base of panel) a brief whine was followed by an arched call and two calls with a slowly falling F_0_. **D** Brief (50–100 ms) high-frequency pulses (12–16 kHz) showing either upward or downward frequency glides with step changes in frequency. These can occur both during and following electrical stimulation (blue line) in juveniles and adults and are distinctive in being very quiet. **E** Call sequence of rising whistles that starts about two seconds after the start of a stimulus train in the PAG of a 39 day old animal. The calls had a rate of about 1.4 Hz.

The main part of the study involved adult animals which were more than six weeks old. However, we also used two young animals, just after they were weaned, with ages of 23 and 27 days old, and three young animals of 33–39 days old, to look for evidence of the distinctive isolation whistle which is produced by infants separated from their mother and siblings. An example of a segment of a natural isolation call sequence from a 16 day old animal, which we collected previously, is shown in Fig 4A, where there are three calls out of a long sequence that lasted for about 20 min. The calls are all rising whistles and variable in their form. The first call in the segment has a brief (about 100 ms) constant frequency component followed by a rapid upwards glide in frequency and the start of an arch where the frequency starts to fall again. The presence of arched calls where the frequency rises and falls is reminiscent of the arched screams of macaque monkeys [23]. The other two calls in the segment have a less steep frequency glide and almost no sign of a fall in frequency at the end. A similar call was produced about 0.4 s after the end of a stimulus train by electrodes placed in the amygdala of a 23 day old animal (Fig 4B). In addition, stimulation in the same animal produced other distinctive calls that we have not observed in adults. These involved an arched call with an F_0_ peaking at just over 2 kHz and calls that start at close to 2 kHz and have descending frequency glides of about 250 ms produced both during and after the stimulus train (Fig 4C). There were also some distinctive high-frequency calls with brief (100 ms) frequency glides in both the ascending and descending direction and sudden steps in frequency within the range of 12–16 kHz (Fig 4D). These calls were produced by stimulation in both juvenile and adult animals, but we have not observed them previously in unstimulated animals.

The isolation whistles are distinctive in that they occur in a regular pattern and can occur continuously for over 20 min while the pup is isolated. In the example shown in Fig 4A, the whistles occur at a rate of 3 Hz and we never saw such a high rate following stimulation. However, we did observe long series of rising whistles/screams and one example is shown on Fig 4E, where a sequence starts about 350 ms after the end of stimulation and continues for just over 16 s. Similar series of regular rising whistles were also produced by stimulating the hypothalamus of a 35 day old animal, although these series were shorter (≤10 s long). Similar series were also recorded when stimulating the PAG and hypothalamus of mature animals, including one of two years old. Thus the sequences of isolation whistles could still be produced by old animals even although they would not be produced under conditions of isolation. We did not record any similar sequences of whistles when stimulating the ACC or amygdala.

### Association of brain structures, weight and gender to particular calls

**Fig 5:**
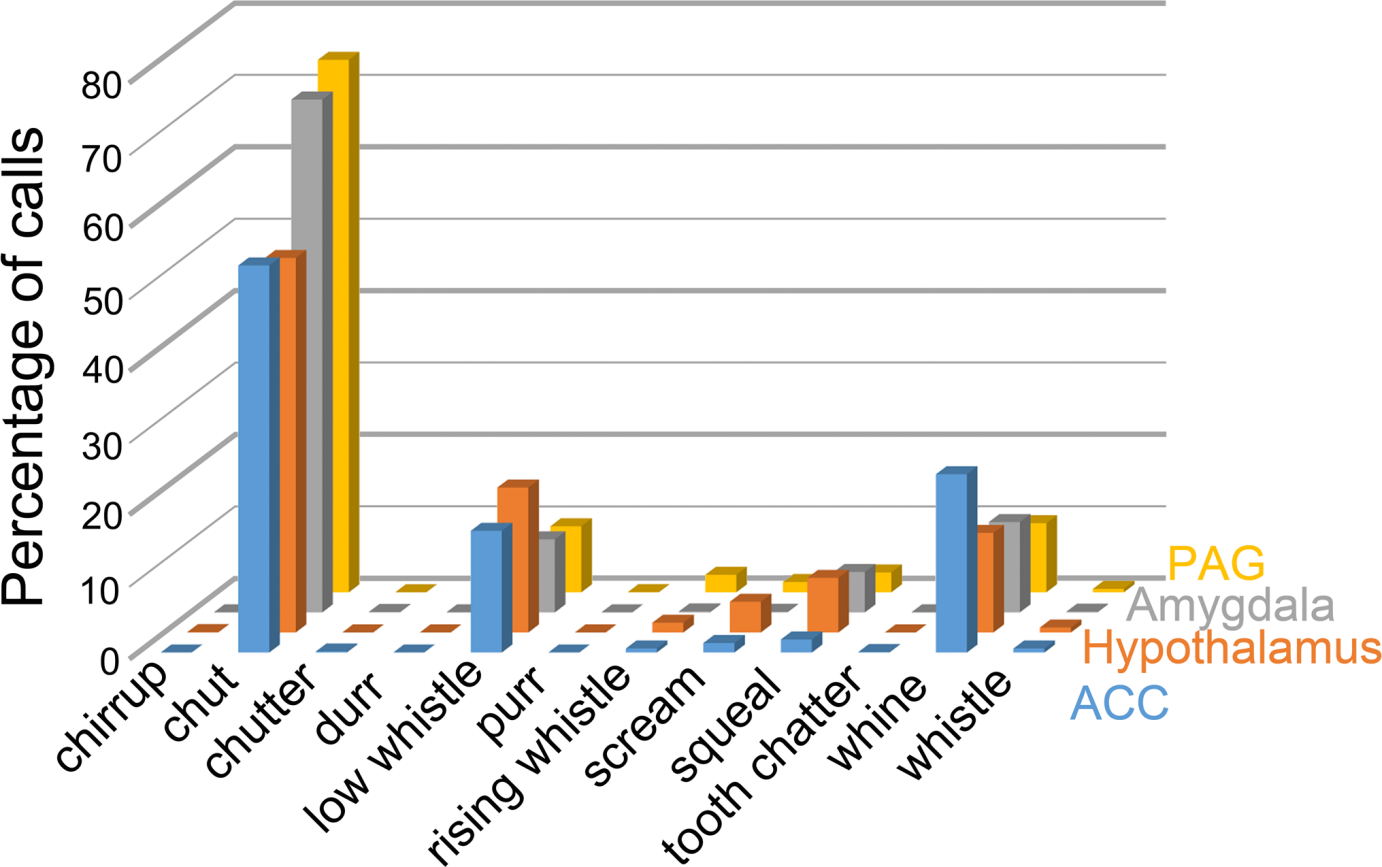
Histogram showing the proportion composed by each call out of the total number of calls produced by each brain structure. The chut is the most common call in all structures and none of the structures produced any chirrup or purr calls. None of the calls appeared to be only associated with one particular structure.

The location of stimulation electrodes was plotted against the calls produced, to look for a correlation. The response changes along an electrode track in any one structure were very variable. Thus some tracks through a structure would typically start with evoking a few chuts and as the electrode went deeper the number of chuts per stimulus train would start to increase and other calls were elicited, so that at a point one mm further down the track there could be a response with five different types of call. However, in some tracks only a variable number of chuts would be produced at all positions along the track where vocalizations were produced. None of the calls was exclusively associated with a particular brain structure, but there were differences in the proportions of calls between structures. The most common call produced in all four structures was the chut which made up between 48 and 63% of the calls produced by each structure. The next most common calls were the whine (8–22%) and the low whistle (8–19%) elicited from each structure. The squeal made up 2–7% of calls for the different structures, while the three high-frequency calls scream, whistle and rising whistle made up less than 4% of calls for any of the structures. The proportions for each call produced by a structure are shown in Fig 5.

**Fig 6:**
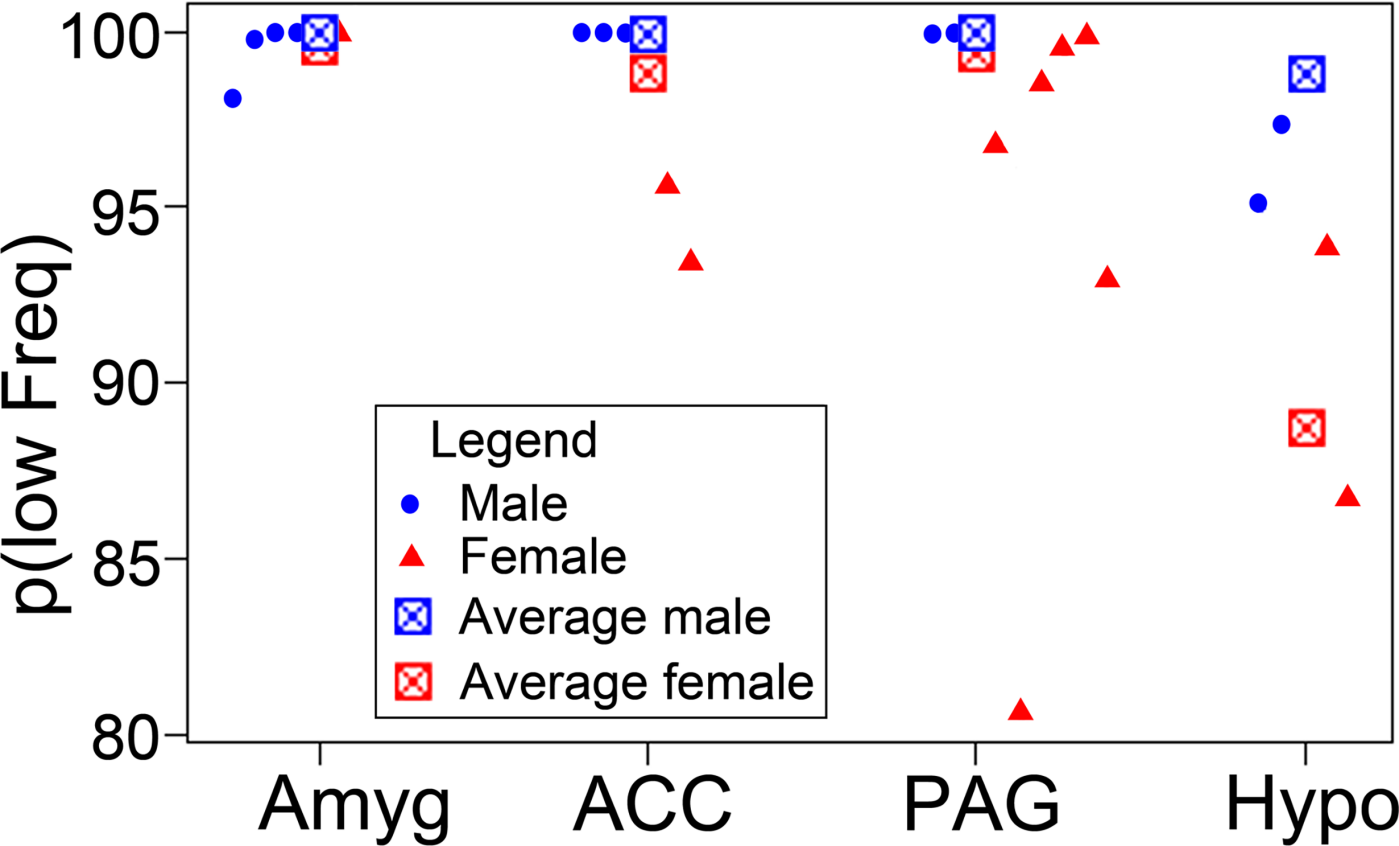
Plot showing the probabilities of obtaining low frequency calls in different animals. Males are indicated by a blue dot and females by a red triangle for the four different structures. The squares show the estimated population-average probabilities for males (blue) or females (red).

There are clear differences in the proportions of calls between different structures but due to the structure of the data and the design of the study a simple chi-square test is not an appropriate way of determining which differences are significant. Instead we used logistic regression implemented in the R package to analyse the data [22]. Logistic regression fits provided statistically significant evidence that the four sites gave different ratios of high- and low-frequency calls (likelihood ratio test χ^2^ = 8.85, Df = 3, p = 0.031). There was also an effect of Gender (χ^2^ = 4.15, Df = 1, p = 0.042), while Weight had no effect overall (χ^2^ = 0.092, Df = 1, p = 0.76). Refitting the model without the Weight variable hardly changed the p-values for the other variables. The gender effect is visible in Fig 6. Female animals (red dots) tend to have lower probabilities. The square symbols show the estimated population-average probabilities as a function of Site and Gender. From the plot it seems that there could be a Site-Gender interaction. However, this cannot be estimated reliably due to the small numbers of animals. A further difficulty for fitting arises from the fact that many of the subject probabilities are very close to one. To double-check the results, the analysis was repeated with a model containing only the Site effect (together with the random effects). It was found that Site remained significant with p = 0.012.

For a further analysis of the Site effect, we performed pairwise comparisons. Technically, this can be done in different ways, for example, by studying coefficients in the models estimated above or by running models that only consider two regions of interest. Both approaches provided similar results. In the model with all four sites and Gender and Weight as covariates there was a highly significant difference between amygdala and hypothalamus (Wald Z-test, β_HT-Amygd_ = -3.45, z = -2.62, p = 0.0087), but no significant difference between amygdala and ACC (β_ACC-Amygd_ = -1.07, z = -0.69, p = 0.49) or amygdala and PAG (β_PAG-Amygd_ = -0.46, z = 0.32, p = 0.75). Using a model containing only Amygdala and Hypothalamus gives a very strong site effect (χ^2^ = 11.84, Df = 1, p = 0.00058) a just significant effect of weight (χ^2^ = 3.95, Df = 1, p = 0.047) and no significant effect of gender (χ^2^ = 0.78, Df = 1, p = 0.38). The gender effect is probably not significant because of the small number of females in this comparison. For some of the other comparisons, results were ambiguous and a larger number of animals would be required to determine if there were significant differences. However, for the model with four sites (and gender and weight) and using the Wald test of coefficients we found: β_PAG-HT_ = -2.99, z = -2.39, p = 0.017; β_ACC-HT_ = -2.38, z = -1.63, p = 0.10 and β_ACC-PAG_ = -0.61, z = -0.43, p = 0.66. Thus there was evidence of a difference between the hypothalamus and PAG, but the difference between hypothalamus and ACC was ambiguous.

In conclusion then, the scream, whistle and rising whistle (high-frequency calls) were produced much less often by stimulation of the amygdala than the hypothalamus. This was particularly true of the scream where stimulation of the amygdala only produced 3 screams in total whereas stimulation of the hypothalamus produced 174 screams.

### Timing of calls relative to the stimulation train

**Fig 7:**
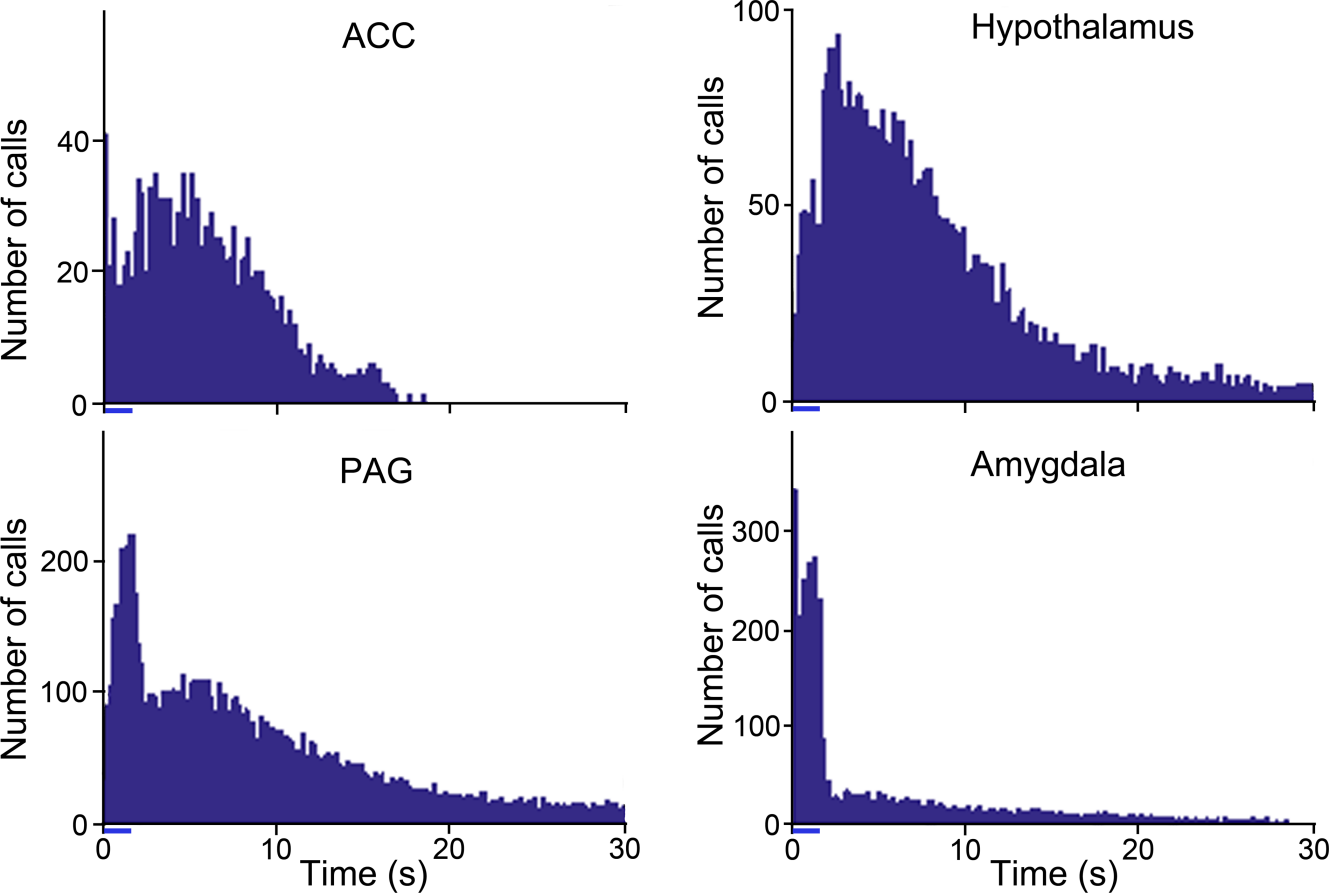
Start times for calls produced by stimulating each brain structure. Histograms showing the times at which individual identified calls of a sequence started relative to the beginning of electrical stimulation. The 1.6 s period of stimulation is indicated by the small blue bar under the x axis. The timings were plotted in 200 ms bins for each structure.

One of the most striking features of the call sequences was that they sometimes continued for a considerable time after the stimulus train had ceased. Usually the call sequence started during the stimulus train, but in some instances, the calls did not start until the stimulus train had finished. In contrast, some call sequences ended as soon as the stimulus train stopped. These different types of response varied between brain structures and an indication of this is given by plotting the start time of all the calls produced by a single structure. The pattern of times are very different as shown in Fig 7 where the number of calls has been plotted in 200 ms wide bins. Stimulation of each structure produces a distinctive pattern with the ACC showing the highest number of calls in the first bin followed by a dip and then a slight increase in numbers after the end of stimulation that gradually tails off so that there are no calls after 20 s. By contrast the hypothalamus produced comparatively few calls in the first 200 ms, but a large number after the end of stimulation which gradually tailed off, but was still present at a low rate after 30 s. This was a very different pattern from the amygdala where there were many calls in the first 200 ms and throughout the stimulation period, but then a sudden, large drop in the number of calls which had ceased by 30 s. The pattern of calls from the PAG was different again with comparatively few calls in the first 200 ms but a peak during the stimulus train and a rapid fall at the end of the train with a gradual tailing off such that there were still some calls after 30 s. We wanted to learn more about why the call patterns were so different and so the next step was to determine how many call sequences were restricted to the stimulation period.

**Fig 8:**
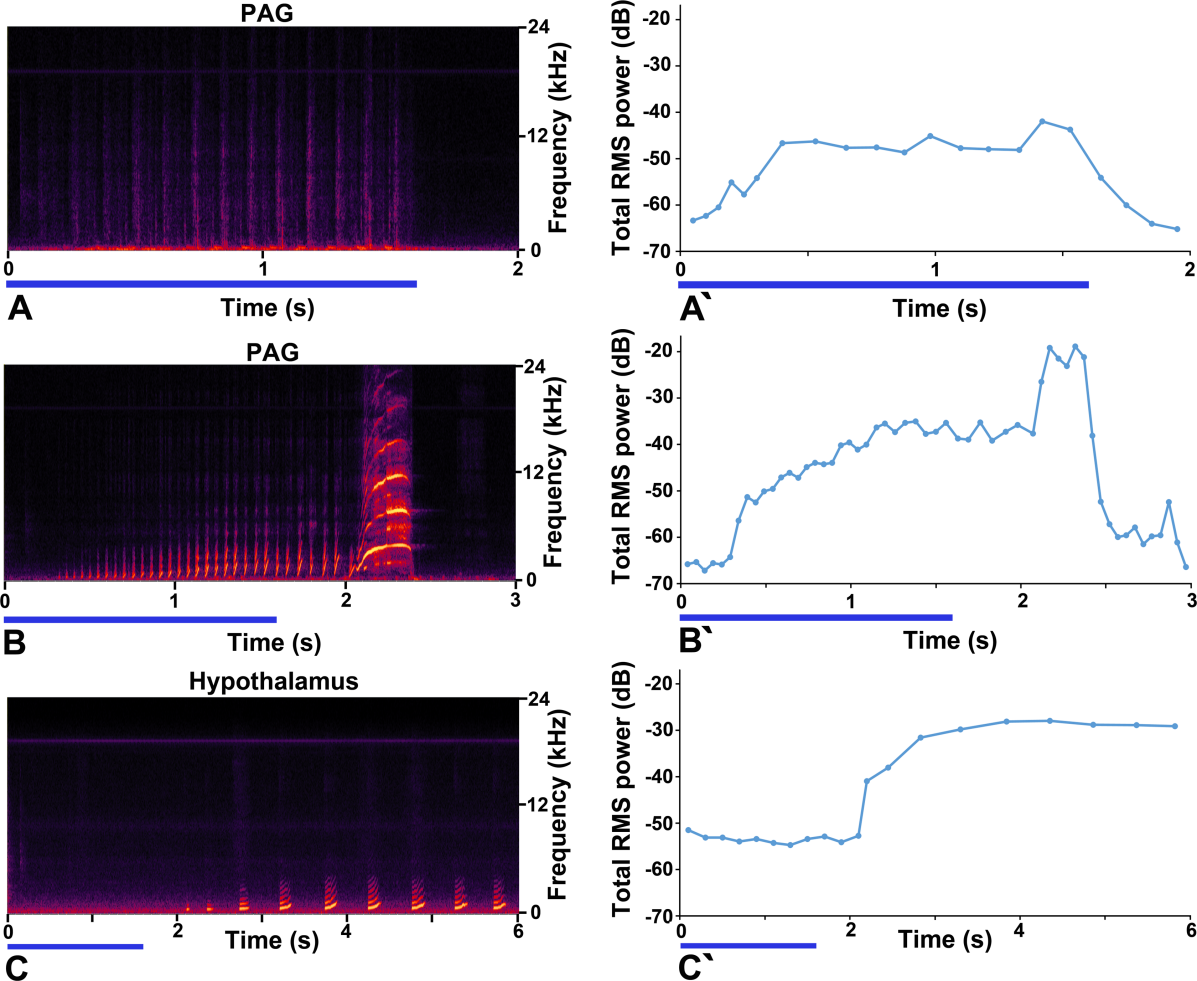
Classification of calls by the temporal position of their sequence. Examples of call sequences with a specific relationship to the stimulus train which is shown by a blue line underneath each panel. **A** “During call”. Stimulation of the periaqueductal grey (PAG) produced a sequence of 13 quiet broadband noise bursts that began and ended within the stimulation period. **A'** The total power in this sequence was measured in 50 ms windows which were centred on the broad band noise bursts when present. The power increased over the first 300 ms and then remained fairly constant until the end of the stimulation period when it stopped abruptly. **B “**Both during and after call”. Stimulation of the PAG also produced a call that started as a series of chuts which extended beyond the stimulation period and came to an end as a rising whistle. **B'** The total power in this call series was measured in 50 ms windows which were centred on the chuts when present. The power in the call increased gradually over the first 1.3 s of stimulation before flattening out and then increasing at 2 s during the rising whistle. **C** “After” call. Stimulation of the hypothalamus produced a series of 14 chuts that started after the end of the stimulation period and continued until 9 s after the start of stimulation, although only the start of this sequence is shown. **C'** The call series does not start until about 500 ms after the end of the stimulation period. The total power was measured in 100 ms windows which were aligned on the start of a chut when one was present. Once the call series starts the power increases rapidly and remains fairly constant until near the end of the series.

**Fig 9:**
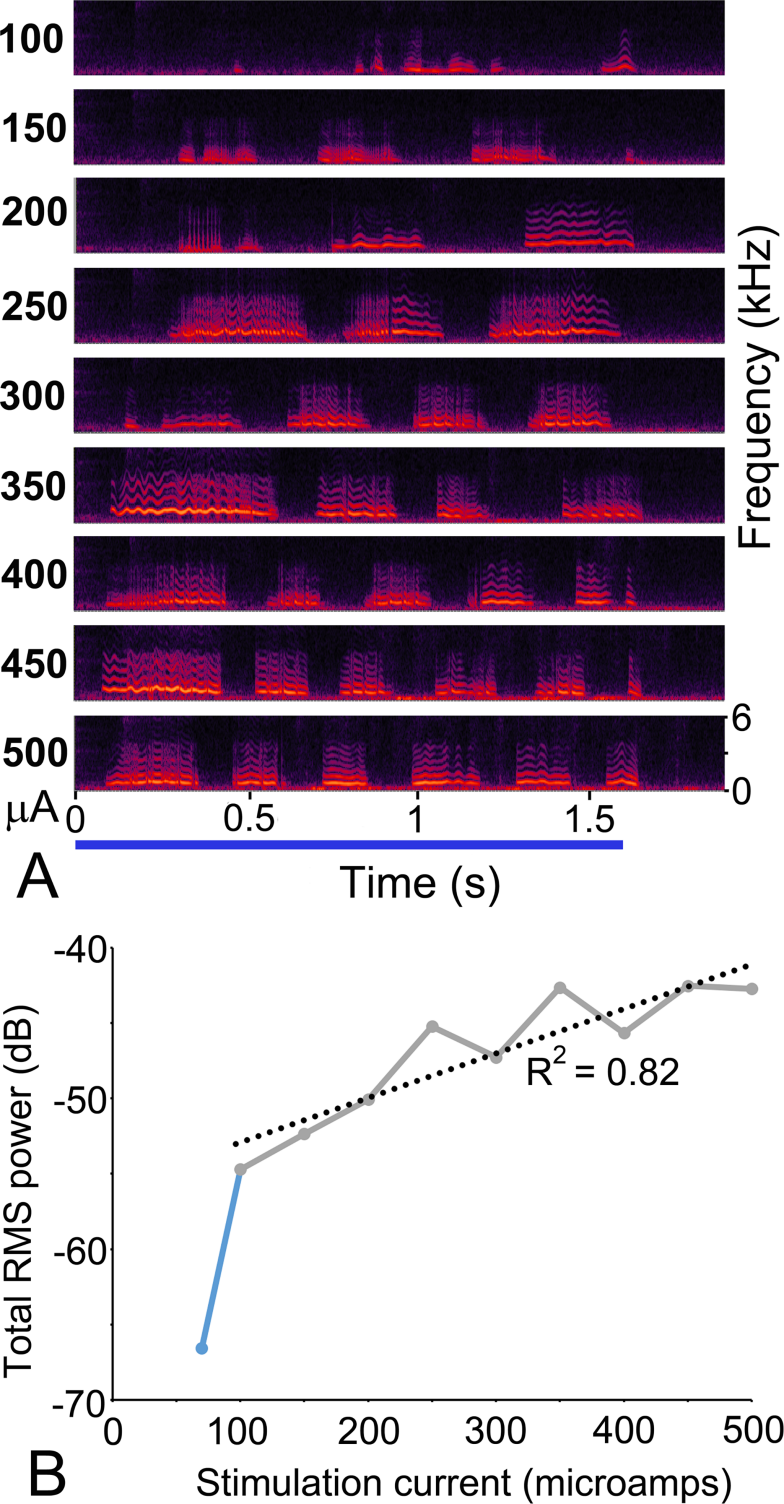
Relationship between stimulation level and power output of calls in the amygdala. A Spectrograms of call sequences produced by stimulating one location in the amygdala at 9 different current levels of between 100 and 500 μA. All the calls were initiated during the 1.6 s long stimulation period (indicated by blue line) and were complete within 100 ms of the end of stimulation. B The total root mean squared power for a window (1.8 s long) covering each call sequence was plotted against stimulation current. The 70μA level was subthreshold and the output power showed a roughly linear relationship to stimulation current when a regression line was fitted to the plot at the higher current levels (dotted line).

We defined three types of call sequences: 1) during stimulus, 2) both during and after the stimulus and 3) after the stimulus train. Examples of all three are shown in Fig 8. In Fig 8A there is a sequence of 13 regular (9 Hz) broadband noise bursts which start about 120 ms after the stimulus train and stop abruptly just before the end of the train. This appears to be a tooth chatter, although it doesn’t correspond to the natural tooth chatter templates that we used. Another example of a “during stimulus” call is shown in Fig 9A. In Fig 8B after a delay of 300 ms there is a regular train of chuts (19 Hz) which continues after the end of the stimulus train when it slows down slightly and changes into a single rising whistle at 400 ms after the end of the stimulus. The main part of the call appeared to be a chutter although it did not correspond to the natural templates that we used. In Fig 8C there were no calls produced during the stimulus period, but a sequence of chuts started 500 ms after the end of the stimulus train.

“During” stimulus calls were mainly observed after stimulation of the amygdala (see Table 2) and we mainly tested the effect of increasing stimulus level on the call sequences in this structure. In the amygdala the majority of call sequences remained of the “During” type even as the current was increased. Out of 67 sites where the current was progressively increased 53 (79%) started as “During” calls at the lowest current levels and 39 of those (74%) remained as “During” calls at all the current levels tested. An example of this call duration stability is shown in Fig 9A where the same site was stimulated at current levels of 100–500 μA. The time at which the last call in the series started was always less than 1.7 s. This temporal stability was not matched by a call stability. These calls did not remain part of an identical series. Instead, each call sequence was produced by a mixture of whines, low whistles and chuts in various combinations. Some of the other call sequences also started out as the “During” type but then changed to the “Both during and after” type as the current increased. This was true in 14/67 stimulated sites (21%), but even in these stimulation series the last call in the sequence never started more than 1 s after the end of the stimulus train. Out of these 14 series the mean time for the last call to start was 2.06 s after the start of stimulation (range 1.719–2.634 s; standard deviation 0.25).

**Table 2:** Numbers and temporal pattern of calls produced by each structure.

Brain structure	No. of calls recorded	No. of call sequences	Mean no. calls/ sequence	No. of “During” sequences (%)	No. of “After” sequences (%)	No. of “Both during and after” sequences (%)
ACC	1414	78	18	3	18	79
Hypo	4097	199	21	0	47	53
PAG	8910	511	17	22	34	44
Amyg	3901	505	8	80	6	14

As indicated in Fig 8 the relationship between the time of stimulation and the time of the maximum power output in the calls could be very different: in some cases there was a complete overlap (“During” calls) and in others a complete phase shift (“After” calls). In addition to this all-or-nothing pattern there were also changes in the power output both during and after the stimulus train. This is illustrated in the right hand panels of Fig 8 which shows three examples of power levels changing. The power in the tooth chatter (Fig 8A' initially increases over the first 400 ms, remains constant and then increases slightly just before the end of the stimulus train before falling fairly rapidly. For other calls, there was a gradual increase in power during the stimulus train, a flattening out and then a sudden increase well after the stimulus had ended. In the case of Fig 8B', the power in the individual chuts increases gradually until about 1.4 s when it flattens out and the power increases again during the rising whistle at the end at least 400 ms after the stimulus had ended. The increase in power after the end of the stimulus train is even more prominent in the “After” stimulus calls as shown in Fig 8C' where the power in the call series increases over the period of about 1 s and then remains reasonably constant until near the end at 9 s after the start of the stimulation.

### Stability of stimulated response at any one electrode position

If the stimulated structures had been motor structures or even pre-motor structures then you would expect that stimulating an electrode at the same position and current repeatedly would give an almost identical call sequence each time. This was only true for a few electrode positions where there was a short and simple call sequence. In most cases, stimulating an electrode at the same position and current repeatedly gave very variable results for repetition, in all four structures. At most locations tested the numbers and often the types of call varied between repetitions. This was not tested systematically and most electrode positions were only stimulated once or twice. A few locations were stimulated with the same current level on 3–5 occasions and this indicated that there were different processes involved in the response lability.

**Fig 10:**
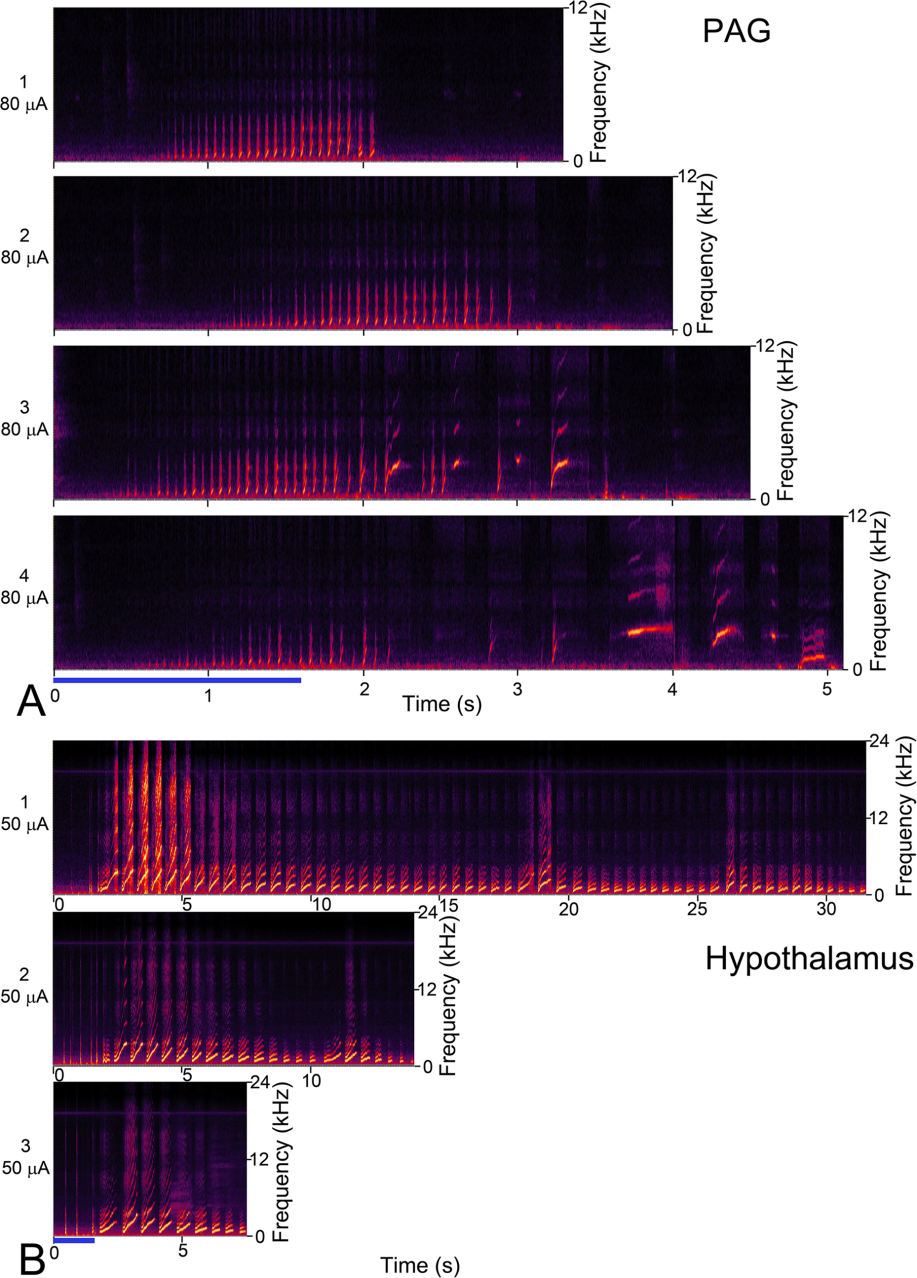
Variability of the response when repeatedly stimulating one electrode at the same position and current level. **A** Spectrograms of call sequences produced by stimulating one location in the PAG at a current level of 80 μA four times in a row. The first two responses to the stimuli train only give a series of rising chuts but the response to the third stimulus train also involves two rising whistles and two incomplete rising whistles (not identified by Avisoft templates). The fourth response includes some incomplete rising whistles as well as two whistles and a low whistle. The duration of the call series increases with each iteration. **B** Spectrograms of call sequences produced by stimulating one location in the hypothalamus at a current level of 50 μA three times in a row. The first response includes 4 chuts, 21 low whistles, 1 rising whistle, 14 screams and 20 whines. The second response includes 12 chuts, 3 low whistles, 3 rising whistles, 3 screams, 2 squeals and 8 whines. The third response includes 5 chuts, 1 low whistle, 3 screams, and 5 whines. Thus the duration of the call series is reduced with each iteration and the number of identified calls changes from 60 to 31 to 14.

At some locations there was evidence of facilitation. This is illustrated in Fig 10A where stimulating four times at one electrode position in the PAG at 80 μA gave four different responses and increasingly long series of calls. On the first occasion, there was a series of rising chuts for the first 2.1 s after the start of stimulation. On the second occasion, the series of rising chuts started later, but went on until about 3 s. On the third occasion the regular series of rising chuts went on to 2.1 s, but then changed into an irregular series of chuts and rising whistles, which continued for about 3.5 s after the start of stimulation. On the fourth occasion, the initial series of rising chuts stopped at about 2.1 s, but then led into some irregular calls that included a whistle, rising whistle, low whistle and more chuts up until 5.1 s.

At other locations there was evidence of habituation. This illustrated in Fig 10B where a single location in the hypothalamus was stimulated three times at a current level of 50 μA. Each of the three call series had different combinations of calls that included chuts, low whistles, rising whistles, screams, squeals and whines. However, both the number of individual calls and the duration of the call series decreased each time the stimulus current was applied. On the first occasion, there were 60 calls over 31.5 s, on the second, 31 calls over 14 s and on the third 14 calls over 7.6 s. The start of the call series was similar each time with a few chuts during the stimulation train followed by a series of 14 screams on the first occasion, but only 3 screams on the second and third occasions.

### Variation in current levels required to produce calls

The final question we wished to answer was whether there was any difference in the ease with which different structures or genders could be stimulated to produce calls. To keep the analysis simple, an average stimulation current was computed for each animal. This was done by taking the mean value for all the different tracks used to sample a structure in each animal and included all tracks that had at least one identified or unidentified call (i.e. tracks containing only artefacts were excluded). The current data were then fitted to an ANCOVA model with factors Site and Gender and covariate Age. We considered that age was a better correlate of neural maturation and degree of myelination than weight. For the fitted model, the test of fixed effects yields a highly significant effect of Site (F(3,16) = 13.58; p = 0.00012) and a significant effect of Gender (F(1,16) = 5.44; p = 0.033), but none of Age (F(1,16) = 0.015; p = 0.90). Fit results changed only very little if Age was removed from the model. There was no evidence of an interaction between Site and Gender. Standard diagnostics did not reveal any substantial problems with the fit. When post-hoc comparisons were made with a Bonferroni test the following values were obtained as shown in Table 3.

**Table 3:** Comparison between mean currents required across structures and gender.

Brain structure (Site)	Mean current (μA)	Std. Error	Pairwise comparison	Mean difference	Std. Error	Significance
ACC	410	36	ACC-Hypo	324	54	0.000
Hypo	85	39	ACC-PAG	235	50	0.001
PAG	175	30	ACC-Amyg	217	50	0.003
Amyg	193	37				
Effect of gender						
M	259	25	M–F	87	37	0.033
F	172	26				

**Fig 11:**
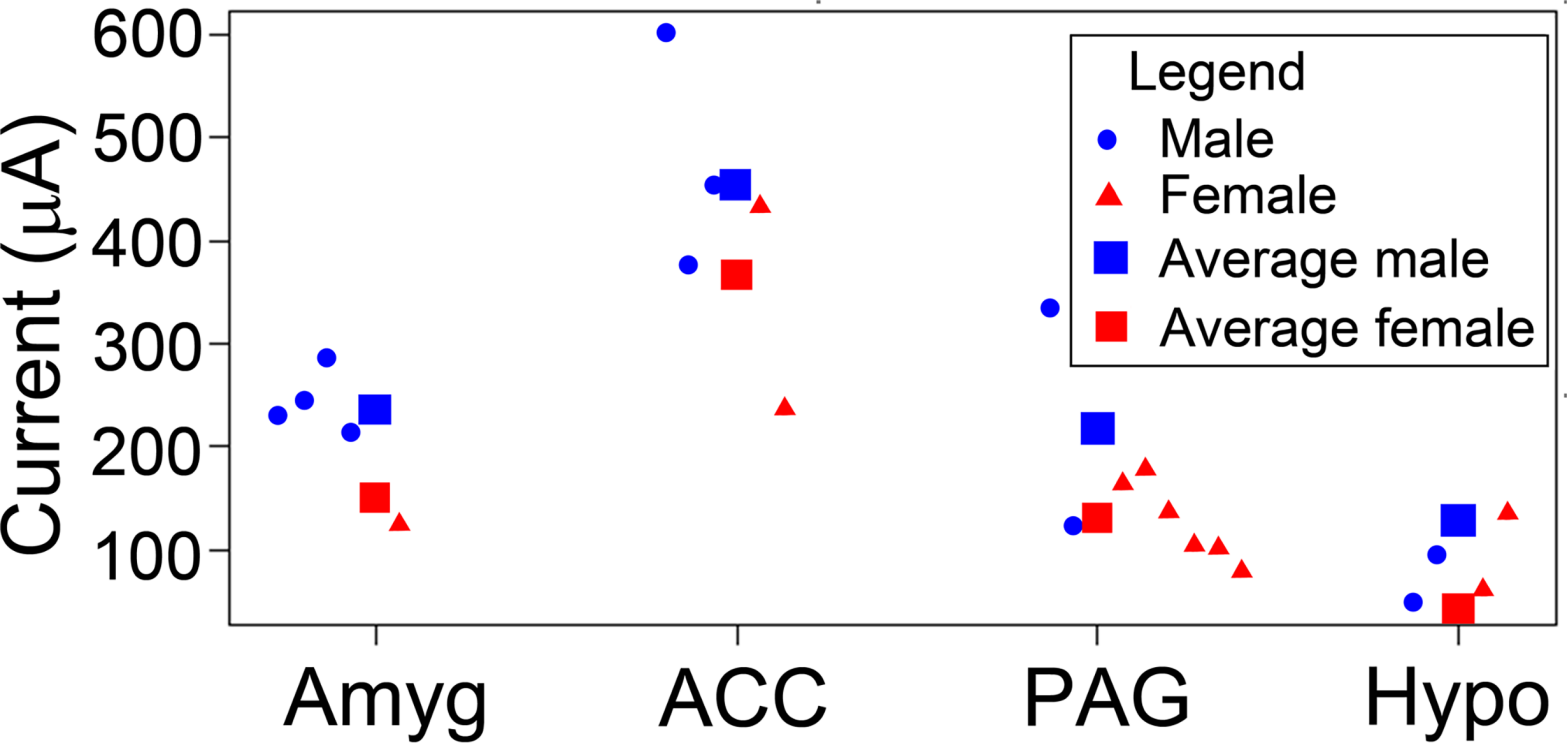
Comparison between genders of the mean stimulation currents. Plot showing the mean stimulation current based on all the tracks for each animal as either a blue dot (male) or a red triangle (female). The mean estimated from the fitted models for each structure is shown as a larger square coloured blue for males and red for females. The mean current for males in each structure is about 90 μA higher than for females.

The ANCOVA results showed that there was no statistically significant differences between the mean currents for Amygdala, Hypothalamus and PAG (Table 3). The strong effect of Site was driven by the difference between these regions and ACC which required a much larger average current. The mean current for male animals when combining all four structures was estimated to be higher by almost 90 μA than for female animals. This is illustrated in Fig 11. This plot shows the observed average currents for individual animals (circles, male; triangles, female) as well as the estimated means (large squares). Blue and red symbols are for males and females respectively. From the plot it might appear that the spread (variance) in currents differs between the Sites. However, a Levene test does not show any statistically significant effect. The Site effect remains significant even if the animal with maximum current in the ACC group is removed from the sample. As further evidence for the robustness of the analysis, it was found that removing tracks with less than 5 or 7 call sequences does not lead to any substantial changes in the results.

### Relationship of power output in the calls to the current level of the stimulation train

**Fig 12:**
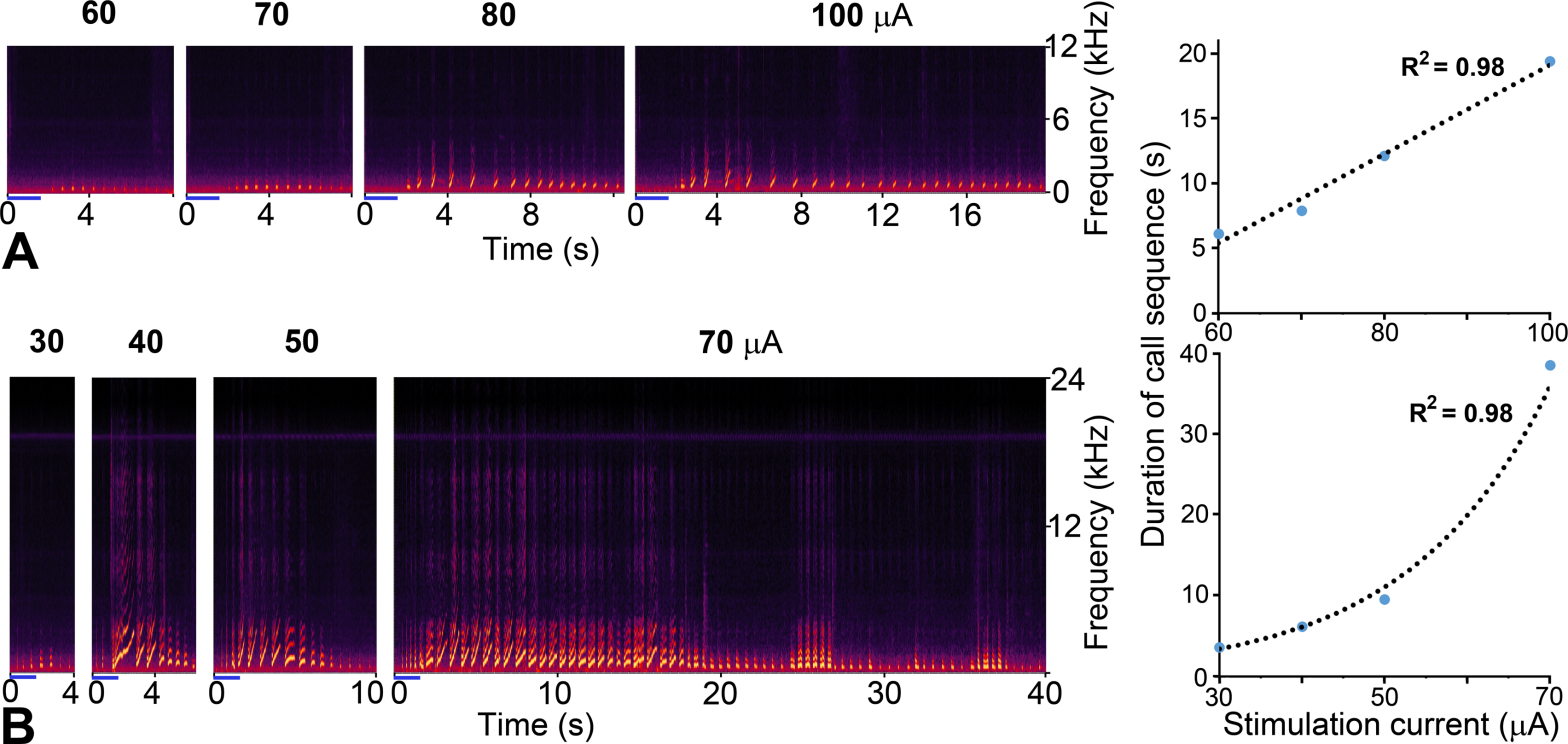
Relationship between stimulation level and power output of calls in the hypothalamus. **A** Spectrograms of call sequences produced by stimulating one location in the hypothalamus at four different current levels of between 60 and 100 μA. All the calls were initiated after the end of the 1.6 s long stimulation period (small blue line) and the sequence continued for increasingly longer periods as the current was increased. The increase in duration was linear over this current range as shown in the right-hand panel where stimulus current is plotted against sequence duration. **B** Spectrograms of call sequences produced by stimulating a different location in the hypothalamus at current levels of between 30 and 70 μA. The calls were initiated during the stimulation period (small blue line) and the sequence continued for increasingly longer periods as the current was increased. The increase in duration was exponential over this current range as shown in the right-hand panel.

Increasing the current level of the stimulation train produced two types of change: one was a roughly linear increase in the power of a “During” call sequence as illustrated in Fig 9B the other was by increasing the duration of the call sequence in the calls that extended beyond the end of the stimulus train. This is illustrated in Fig 12 where two different sites in the hypothalamus were each stimulated at four different current levels. At one site (Fig 12A) there was a linear increase in the duration of the call sequence with increasing current, while at the other there was an exponential increase (Fig 12B). The sites showing a linear increase in call duration with increasing current were more common in the hypothalamus with 7/9 (78%) sites showing a linear increase and 2/9 (22%) showing an exponential increase. Four sites in the ACC which were tested for the effect of varying stimulation current also showed a linear increase in duration and these were either “After” or “Both during and after” types. However, in the PAG, these types showed no consistent relationship between current level and sequence duration. At two sites there was a decrease in sequence duration with increasing current, at three sites there was a linear increase and at four there was an increase and then a decrease in sequence duration with current increase.

## Discussion

### Definition and identification of guinea pig calls

Domestic guinea pigs are very vocal animals and particular calls have been associated with certain behavioural situations: for example, the rut rumble (purr) with mating behaviour [5] and the tooth chatter with aggressive encounters [9]. The behavioural context has been identified for another ten identified calls [6]. However, these early studies made it clear that the variety within any one call type and the overlap between them meant that it was difficult to produce precise definitions that would allow individual calls to be ascribed to a distinct class. The presence of hybrids and intermediate forms meant that calls appeared to be gradually modified and shifted from one class to another [24]. This gradual shift was clearly present in some of the call sequences present following electrical stimulation. It was especially evident among the low-frequency calls of squeal, whine, low whistle and chut where the main distinguishing feature was the duration of the call. The four calls tended to be intermingled, in a variety of combinations in any one call sequence, both among themselves and the high-frequency calls. Despite this the identification of about 12 innate vocalizations was common across a variety of South American rodent species [25] and in some primate species such as the marmoset [26] and human [27]. GPs only show a very limited ability to learn to modify their calls and the ability to produce their calls is almost certainly innate. They are able to produce normal isolation whistles even when their cochleas have been destroyed one day after birth [28]. Studies in other vocal non-learning animals has revealed that deaf animals, or those reared in isolation or by mute mothers, are still able to produce all of the vocalizations used by normal animals. Humans too, have a repertoire of innate vocalizations. Some of these (such as ‘cry’) are present from birth [29], whereas others develop over the first 3 months of life. With one exception (babbling) of their twelve vocalization types, there are no differences between normal hearing and hearing impaired babies in the spectral properties of their calls or the age at which they appeared [30, 31].

### Similarity between electrically stimulated and naturally produced vocalizations

While in the main the electrically evoked calls were very similar to spontaneous calls, some differences remain. It is difficult to draw firm conclusions from our study about why rhythmic calls such as purr and chirrup were not produced by electrical stimulation. One source of differences between the stimulated and spontaneous calls is probably the general anaesthesia. A surgical level of anaesthesia abolished the calls altogether and the presence of an anaesthetic can produce radical changes in the way that a call is represented in the auditory cortex [32]. However, even in conscious squirrel monkey electrical stimulation was only able to produce 11 out of the 26 innate calls that have been described under natural conditions [33]. Monkeys also produced calls under light general anaesthesia that appeared similar to those produced by the awake animal [34]. Extensive work on the chicken showed that the type of call produced at a certain location in an awake bird did not change when it was under general anaesthesia [35]. However, the presence of a general anaesthetic would be expected to affect the spectral and or temporal aspects of the calls produced by electrical stimulation. Thus in isolated guinea pig pups, high-pitched distress calls are emitted which have a dominant frequency of between 2.2 and 7 kHz in awake animals [7] while the corresponding calls produced by electrical stimulation of the PAG in anaesthetised guinea pigs have frequencies that are barely half this [15]. We did observe series of rising whistles that resembled the isolation calls except that the pulse rate was slower than that produced by isolated pups. The study by Kyuhou and Gemba [15] also described calls which were identified as purrs, but had upwardly sloping fundamental frequency trajectories that did not look like the flat pulses of sound described in the short purrs of young animals of a similar age [8]. In a similar study of urethane anaesthetised guinea pigs, stimulation of the PAG did not produce any calls that could be identified as either short or long purrs [16]. Similarly in our study, there were a few calls that resembled short purrs, but their fundamental frequencies were higher and their pulse rate was lower than those for natural purr calls. Thus only two out of over 20,000 call pulses were identified as the short purr (durr). We never identified any chirrup sequences either. We did observe series of broadband noise bursts that seemed to be caused by teeth chattering, but these series of pulses were always slower than our templates of naturally occurring tooth chatters. Similarly, we saw regular series of chuts following electrical stimulation, but the pulse rates were slower than for our naturally produced chutter templates. Thus, we concluded that all of the rhythmic natural calls that occurred in regular sequences were different following electrical stimulation. Most of these call sequences would mainly have occurred after the end of stimulation and this implies that their alteration was due to an effect of the anaesthesia on the call generators in the pons and medulla. Our syllable based analysis system [36] was too rigid to make allowance for altered temporal patterns. By contrast, all of the calls that occurred individually or as a small group were very similar to the natural calls and were routinely matched to the natural templates by the Avisoft programme.

### Identification of new calls

Out of the 20,977 calls analysed in this study 661 (3.15%) were not identified as either similar to one of our natural templates or artefacts (possibly tooth chatters). These unidentified calls were not studied systematically although many of them did seem similar to the naturally occurring calls despite not reaching the 0.65 correlation value with our range of templates. Thus there were calls (Fig 4C) that appeared similar to the arched and falling frequency glides that have been described in some isolation whistles of isolated pups [7]. However, a few of the calls did not look like the types of calls that have been described previously by ourselves or others in the guinea pig. These were brief, tonal calls with either rising or falling frequencies that sometimes showed abrupt steps in frequency and had energy in the range of 12–16 kHz. They were similar to the stepped ultrasonic vocalizations that have been described in mice [37, 38] except that the mouse calls are in the range of 40–90 kHz. These calls are most common during mating activity, particularly the mounting phase [39]. We have not studied mating behaviour in guinea pigs and although the calls are not ultrasonic, their high frequencies and relative quietness would make them very difficult to detect without recording equipment. This may be why they have not been detected in previous studies.

### Contribution of individual brain structures to a particular call

In early mapping studies of the brain different areas were systematically mapped by inserting electrodes and stimulating at all sites along a track in awake animals. These studies involved monkeys such as the macaque [40] and squirrel monkey [33] as well as non-primates such as the rat [41] and guinea pig [12]. The studies gave a good indication of which structures were involved in producing vocalizations and emphasised that they were widespread and generally associated with the limbic system and brainstem particularly those parts that were involved in the reward system and self-stimulation [41]. However the studies did not always have access to specialised sonographic equipment that would have allowed them to make an objective spectrographic analysis of the calls. By the time modern digital equipment was available interest had turned to the production of guinea pig calls as a sensitive assay to screen for the effectiveness of anxiolytic drugs in reducing the isolation stress shown by young pups [42, 43] or in reducing nociception [44]. Thus this seems to be the first study in the guinea pig to attempt a comprehensive spectrotemporal analysis of the calls along with the stimulation of multiple brain structures.

The mapping studies had emphasised the complex nature of the call-producing systems and that multiple structures were involved in the production of any one call. Despite this there were suggestions that particular limbic structures had a distinct role in the production of calls such as the isolation whistle. Thus the ACC was thought to be involved in gating the production of most innate calls [18] but particularly the pup isolation whistle [45]. The basolateral amygdaloid nucleus was also thought to be involved in the production of isolation whistles in guinea pig pups through the release of substance P into the amygdala caused by the increased stress [46]. More recent work has emphasised the distributed nature of the neural networks that are involved in suppressing the number of vocalizations following pup isolation. Thus nociception opioid receptor (NOP) agonists strongly reduced the total number of vocalizations produced by the isolation of guinea pig pups [43] and the NOP receptors are widely distributed throughout the brain, particularly in limbic system structures such as the amygdala, PAG and hypothalamus.

Our results showed that a similar range of calls could be produced by stimulating all four structures and again emphasised the distributed nature of call production circuitry. The spectrographic structure of the calls didn’t seem to have any direct link to their behavioural meaning as the effect of any call seems to be very dependent on the context. Berryman [6] gave a comprehensive description of the behavioural context of all the guinea pig calls. She showed that the short purr (durr) is spectrally identical to the long purr and yet the short purr is a mild warning or alarm call, while the longer purr seems to indicate a desire for close physical contact [6]. In our study, both proximity maintaining/regaining calls such as chut and low whistle were often produced in the same call series as the aversive calls of whine and squeal. Similarly, the rising whistle and scream are spectrally similar calls, but the whistle is associated with proximity regaining, while the scream is an aversive call that may denote pain. Despite this, both calls were sometimes produced in a single series by stimulating at one site. However, the very low frequency with which screams (denoting pain) were produced by amygdalar stimulation ties in with previous work showing that stimulation of the central amygdala promotes antinociception [36]. This indicates that there was some link between the function of a brain structure and the calls it is involved in producing most frequently.

On the other hand, the ventral hypothalamus was thought to have a primary role in initiating purring in cats [47] and the mating purr in guinea pigs [15]. We were unable to elicit the purr call from our anaesthetised guinea pigs and couldn’t assess the role of the hypothalamus in producing this call. In our results, the more striking finding was the relatively large number of screams that were produced by stimulating this structure. This ties in with the role of the hypothalamus in producing pain vocalizations. The ventromedial hypothalamus is thought to be a core structure in generating affective behaviour in response to threats. Stimulating it in rats produces vocalizations that are similar to those produced by the noxious tail-shock [48]. The PAG also has a central role in vocalization behaviour [49] and there are sub-regions within it that are associated with distinct calls [15, 50]. We saw little evidence of unique calls from distinct sub-regions in the guinea pig. Most sites produced a number of chuts and these could be followed by examples of between one and six other call types. However it was true that some tracks were more strongly associated with high-frequency calls than others.

The hypothalamus has many sub-nuclei and the amygdala has at least four divisions each of which may have a different role in call production or suppression. However, our very basic histology, the relatively large current spread from our bipolar electrode arrangements and the possibility of stimulating fibres of passage meant that our experiments were not designed for distinguishing between the roles of different sub-nuclei. The basic Nissl stain was not suitable for reliably defining the borders of sub-nuclei and the current levels we used could potentially activate thousands of neurons [51] as increasing the stimulation current leads to an increased area of activated neurons. To avoid fibres of passage we would have had to use neurochemical stimulation to increase neurotransmitter levels such as glutamate. In addition to activating cell bodies exclusively, these techniques also provide information as to the specific neuron type responsible for vocal production [52–55]. Our experiments were designed to analyse the types of call produced by each structure rather than to assess the contribution of different sub-divisions.

### Relationship of time and intensity of stimulation to call production

The separation of calls into “During” and “After” stimulation types was very striking. This dichotomy of call types had been described in awake animals such as the macaque monkey [40] and the chicken [35], but had not been specifically studied previously in the guinea pig when either awake [12] or anaesthetised [15–17], where the described calls were present both during and after the same stimulation train. Despite the presence of this hybrid form we were clearly able to distinguish calls that were either exclusively “During” or exclusively “After”. Even increasing the levels of stimulation usually did not change one of these call types into the hybrid form of “Both during and after” calls. Instead the increased current would sometimes either increase the intensity of the call without affecting its duration or increase its duration without having much effect on its intensity. The presence of “During” calls occurring within a short latency of the start of stimulation did not indicate that these were direct pre-motor centres. The pattern generators for controlling the complex muscle responses responsible for producing the vocalizations are located in the reticular formation of the pons and medulla [1]. Structures in the limbic system have a direct projection to the reticular formation and the neurons in and around the nucleus ambiguus [56]. Both the limbic system structures and the PAG all seemed to belong to complex circuits involved in call production and the latency between the start of the stimulus train and the first call partly depended on whether the animal inspired first before calling [57] or went straight into a call without a preceding inspiration [58]. Our results are consistent with the conclusions of Robinson [40] who stated that the production of innate vocalizations depends on, “a highly complex system, widely distributed to all parts of the limbic system, in which geometrical simplicity plays no part and offers no explanatory schemata”.

### Effect of gender, age and size on call production

We saw a clear effect of gender on both the types of calls produced and the ease with which they could be elicited. Although the effects were fairly small the large numbers of calls analysed meant that the effects appeared to be quite robust. Following stimulation of the ACC, PAG and hypothalamus there was a higher probability of eliciting a high-frequency call from the females than the males. The mean current used to elicit all of the calls was also significantly lower in the females and we interpreted this as indicating that the brainstem pattern generators in females had a lower threshold for activation than in males. This implied that females were more likely to produce calls and especially high-frequency calls than males under the same conditions when awake. Gender differences have been described in behavioural studies of other species as female spider monkeys are more vocal than the males in the wild [59] and in macaque monkeys infant males are less likely to vocalise and they show a smaller range of separation calls than females [23]. These differences can be modified by prenatal androgens. In rats there is also some evidence of females normally producing more spontaneous short ultrasonic vocalizations than males [60] although, in infant guinea pigs, males vocalized more than females during isolation [61]. These gender differences may be underpinned by anatomical differences in the medial amygdala [62] and the hypothalamus [63] which are linked to gender.

There was a weak but significant effect of age/size on some of the vocalizations produced. Stimulation in young animals was likely to give more high-frequency calls than stimulation of the same structures in older animals. Again this was expected as the pup-isolation whistles are relatively high-frequency and in awake animals are much more likely to be produced by an infant than a mature animal [7]. As guinea pigs grow and mature their larynx and vocal pathway grow longer and this lowers the fundamental frequency of calls like the purr [8]. However we didn’t perform a fine-grained analysis of variation in the fundamental frequency on individual call types and would probably not have picked up changes of this type.

Our main conclusions are that electrical stimulation of various structures in the anaesthetised guinea pig brain can reliably produce at least seven types of short call, that are very similar to those in the awake animal, but that the rhythmic calls such as chirrup and purr are not produced. The temporal pattern of calls in relation to the stimulation train varied between areas and the female brains were more easily stimulated and more likely to produce high-frequency calls.

## Supporting information

S1 Table The ARRIVE guidelines checklist for reporting animal data. (PDF)
